# Supplementary material for: High density cultivation for efficient sesquiterpenoid biosynthesis in Synechocystis sp. PCC 6803
Source: Sci Rep. 2020 Apr 3;10:5932. doi: 10.1038/s41598-020-62681-w (PMC7125158; doi:10.1038/s41598-020-62681-w)
Supplement: Supplementary file 1 — Supplementary Information. [file 41598_2020_62681_MOESM1_ESM.pdf]

High density cultivation for efficient sesquiterpenoid biosynthesis in *Synechocystis* sp. PCC 6803

Dennis Dienst[1], Julian Wichmann[2], Oliver Mantovani[1], João S. Rodrigues[1], Pia Lindberg[1*]

[1]Department of Chemistry – Ångström, Uppsala University, Uppsala, Sweden

[2]Faculty of Biology - Center for Biotechnology, Bielefeld University, Bielefeld, Germany

*Correspondence should be addressed to Pia Lindberg (email: pia.lindberg@kemi.uu.se)

Supplementary Figures

a

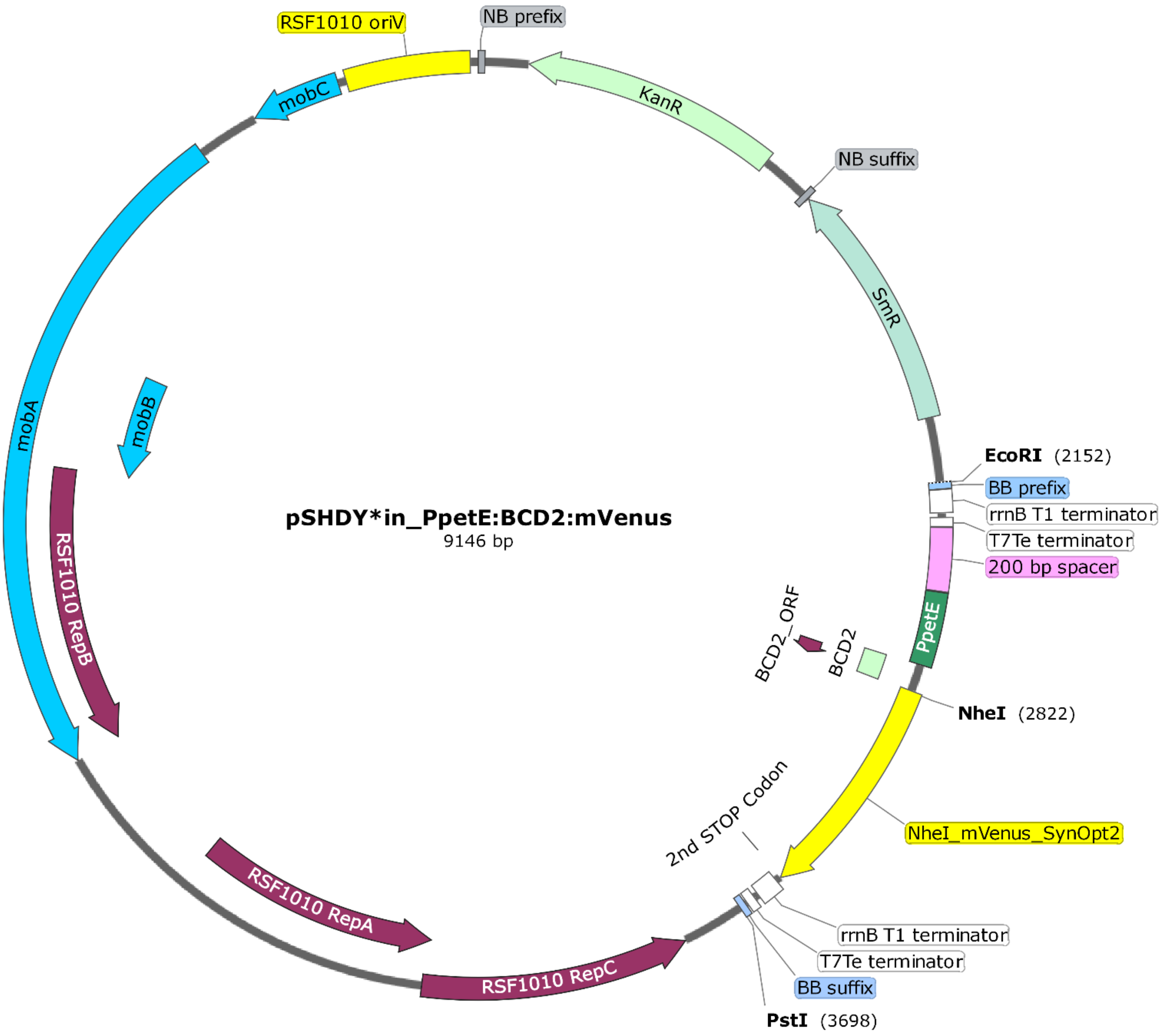

RSF1010 oriV
NB prefix
mobC
KanR
NB suffix
SmR
mobB
mobA
pSHDY*in_PpetE:BCD2:mVenus
9146 bp
RSF1010 RepB
EcoRI (2152)
BB prefix
rrnB T1 terminator
T7Te terminator
200 bp spacer
PpetE
BCD2_ORF
BCD2
NheI (2822)
2nd STOP Codon
NheI_mVenus_SynOpt2
RSF1010 RepA
RSF1010 RepC
rrnB T1 terminator
T7Te terminator
BB suffix
PstI (3698)

b

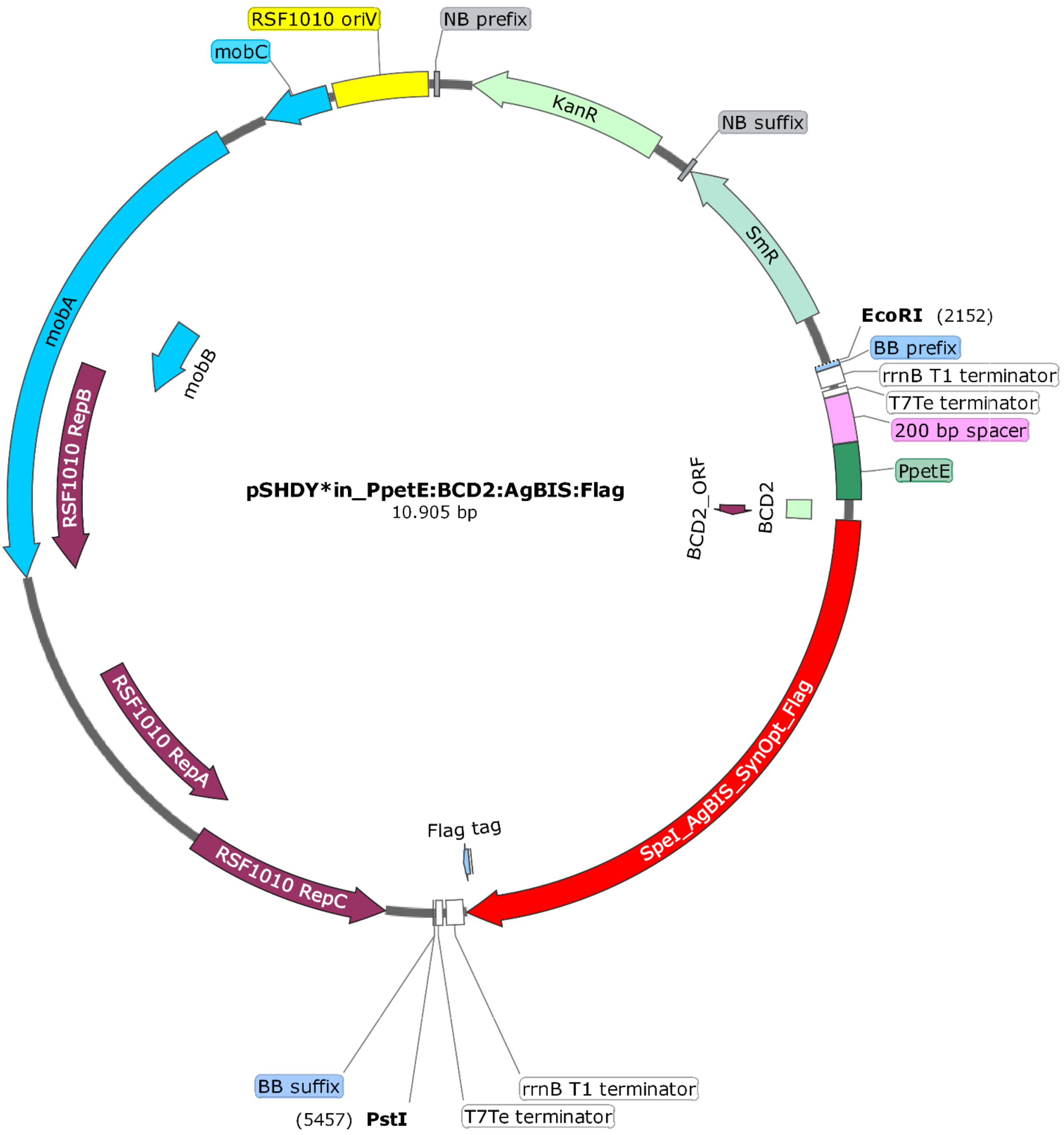

RSF1010 oriV
NB prefix
mobC
KanR
NB suffix
SmR
EcoRI (2152)
BB prefix
rrnB T1 terminator
T7Te terminator
200 bp spacer
PpetE
mobA
mobB
RSF1010 RepB
pSHDY*in_PpetE:BCD2:AgBIS:Flag
10.905 bp
BCD2_ORF
BCD2
RSF1010 RepA
SpeI_AgBIS_SynOpt_Flag
Flag tag
RSF1010 RepC
BB suffix
(5457) PstI
rrnB T1 terminator
T7Te terminator

c

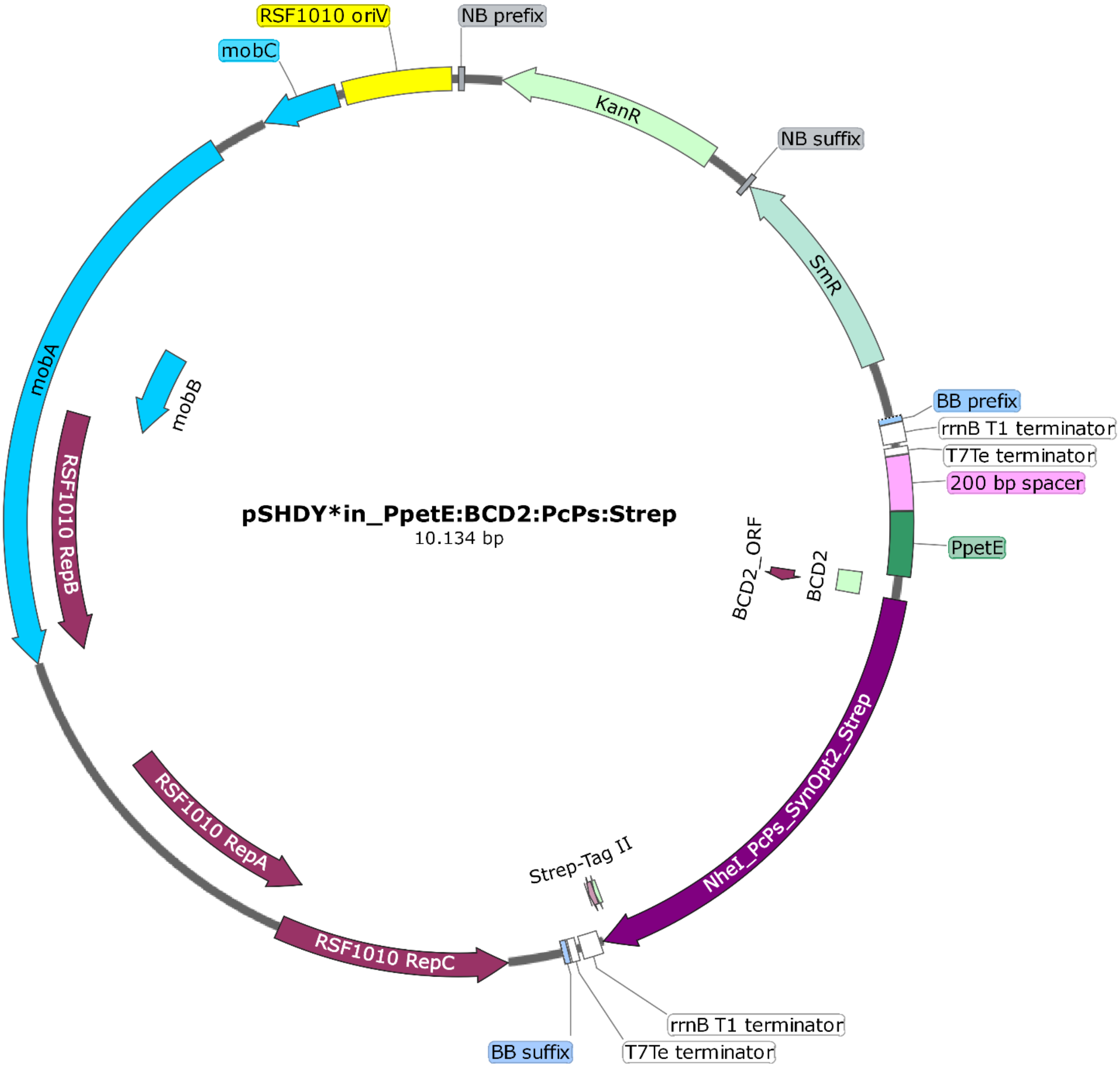

RSF1010 oriV
NB prefix
mobC
KanR
NB suffix
SmR
BB prefix
rrnB T1 terminator
T7Te terminator
200 bp spacer
PpetE
BCD2_ORF
BCD2
mobA
mobB
RSF1010 RepB
pSHDY*in_PpetE:BCD2:PcPs:Strep
10.134 bp
NheI_PcPs_SynOpt2_Strep
Strep-Tag II
RSF1010 RepA
RSF1010 RepC
rrnB T1 terminator
T7Te terminator
BB suffix

d

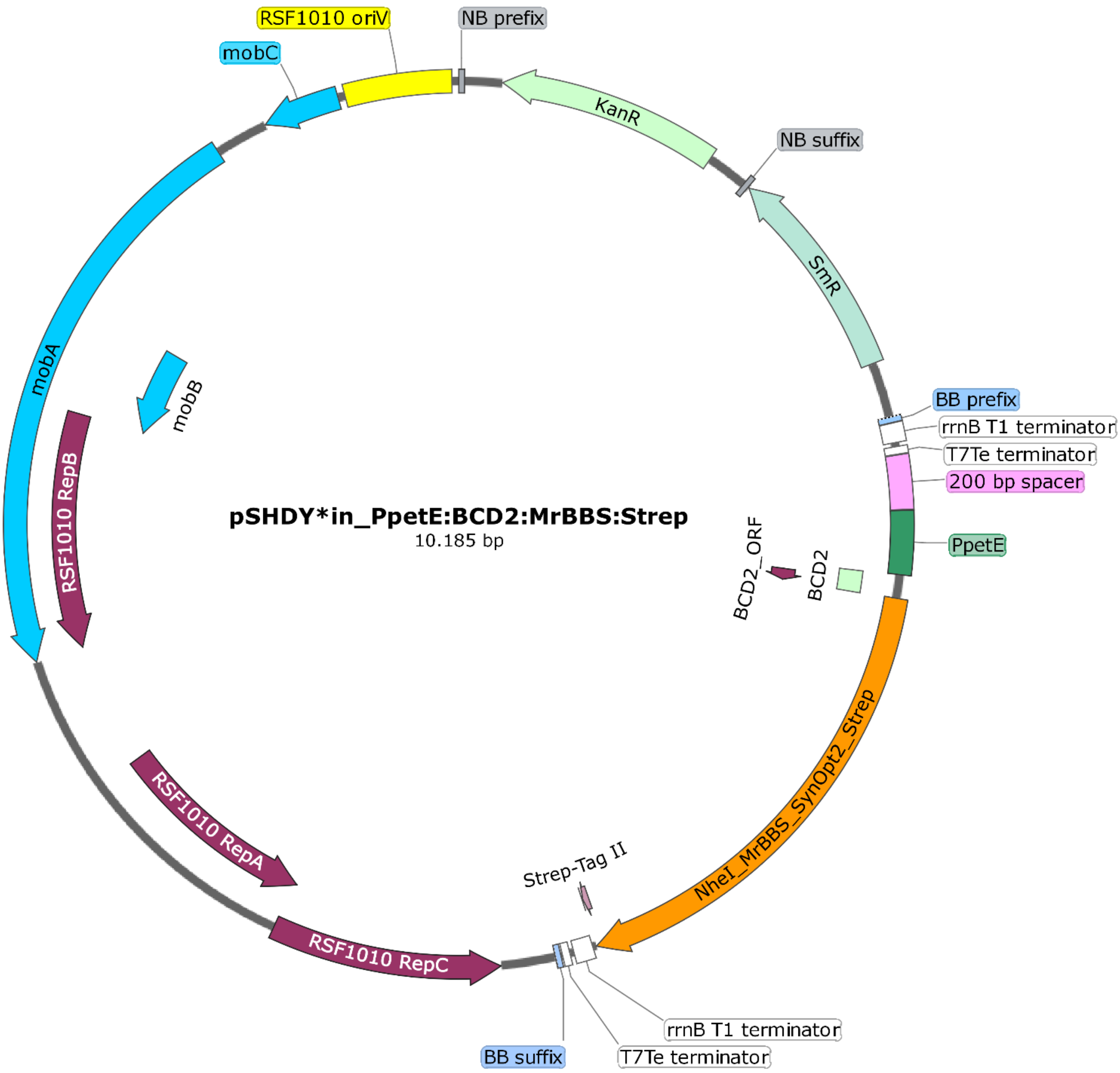
pSHDY*in_PpetE:BCD2:MrBBS:Strep
10.185 bp
RSF1010 oriV
NB prefix
mobC
KanR
NB suffix
SmR
BB prefix
rrnB T1 terminator
T7Te terminator
200 bp spacer
PpetE
BCD2_ORF
BCD2
NheI_MrBBS_SynOpt2_Strep
Strep-Tag II
rrnB T1 terminator
T7Te terminator
BB suffix
RSF1010 RepC
RSF1010 RepA
RSF1010 RepB
mobA
mobB

e

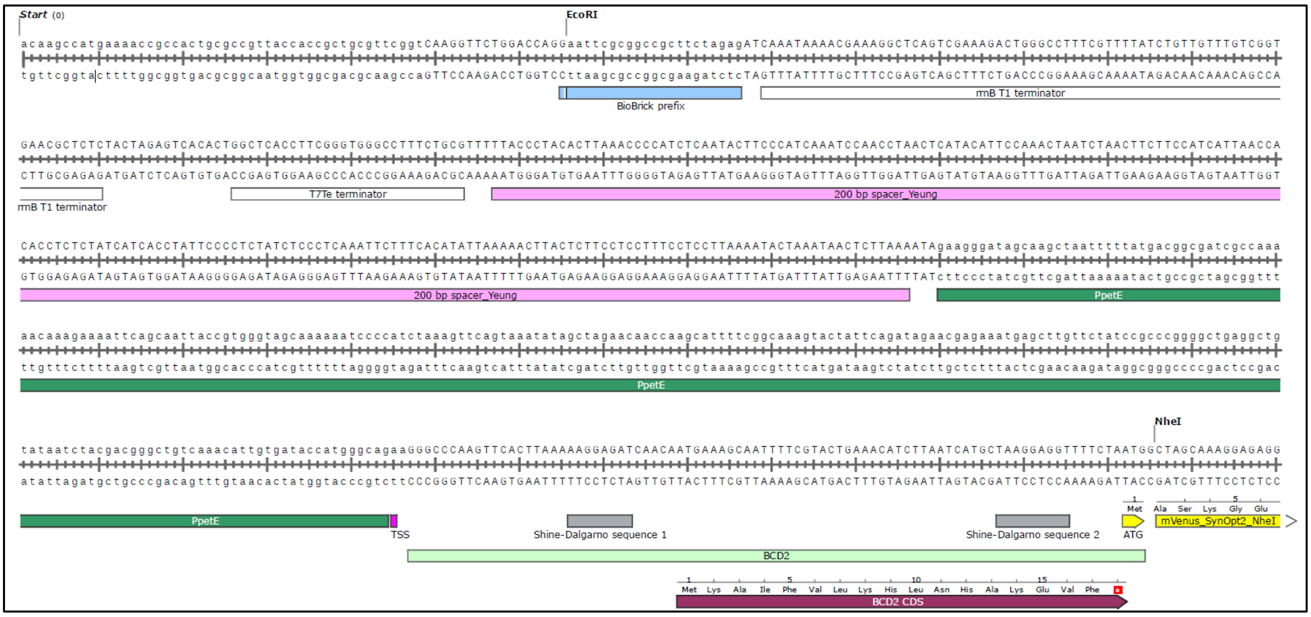

f

Feature	Sequence	Reference
BioBrick prefix	GAATTCGCGGCCGCTTCTAGAG	http://parts.igem.org/Part:BBa_G00000
double terminator B0015 (rrnB T1/ T7Te)	ATCAAATAAAACGAAAGGCTCAGTCGAAAGACTGGGCCTTTCGTTTTATCTGTTGTTTGTCGGTGAACGCTCTCTACTAGAGTCACACTGGCTCACCTTCGGGTGGGCCTTTCTGCGTTT	http://parts.igem.org/Part:BBa_B0015
200 bp spacer	TTACCCTACACTTAAACCCCATCTCAATACTTCCCATCAAATCCAACCTAACTCATACATTCCAAACTAATCTAACTTCTTCCATCATTAACCACACCTCTCTATCATCACCTATTCCCCTCTATCTCCCTCAAATTCTTTCACATATTAAAAACTTACTCTTCCTCCTTTCCTCCTTAAAATACTAAATAACTCTTAAA	Yeung et al., 2017[1]
P*petE* promoter region<br>*TSS according to Mitschke et al. (2011) in boldface	GAAGGGATAGCAAGCTAATTTTTATGACGGCGATCGCCAAAAACAAAGAAAATTCAGCAATTACCGTGGGTAGCAAAAAATCCCCATCTAAAGTTCAGTAAATATAGCTAGAACAACCAAGCATTTTCGGCAAAGTACTATTCAGATAGAACGAGAAATGAGCTTGTTCTATCCGCCCGGGGCTGAGGCTGTATAATCTACGACGGGCTGTCAAACATTGTGATACCATGGGCAG**AA**	Mitschke et al., 2011[2]
Bicistronic design *BCD2	GGGCCCAAGTTCACTTAAAAAGGAGATCAACAATGAAAGCAATTTTCGTACTGAAACATCTTAATCATGCTAAGGAGGTTTTCTAATG	Mutalik et al. 2013[3]

Figure S1*:* Map of vectors (a) pSHDY*in_PpetE:BCD2:mVenus, (b) pSHDY*in_PpetE:BCD2:AgBIS:Flag, (c) pSHDY*in_PpetE:BCD2:PcPs:Strep and (d) pSHDY*in_PpetE:BCD2:MrBBS:Strep used for reporter fluorescence monitoring (a) and heterologous biosynthesis of sesquiterpenes (b, c, d) in *Synechocystis*, respectively. Insertion of the constructs "MrBBS:GS:Strep: T1/ T7Te " and "PcPS:GS:Strep: T1/ T7Te " into the pSHDY_in_PpetE:BCD2 backbone was conducted via NheI ($2^{nd}/3^{rd}$ codon of CDS) and PstI (downstream of double terminator rrnB T1/ T7Te), using pSHDY-in_PpetE:BCD2:mVenus as cloning template. Construct "AgBIS:AGS:Flag:T1/ T7Te" was inserted via SpeI and PstI, thereby destroying the compatible NheI site in the template. Gene expression in *Synechocystis* was mediated by the native copper-inducible promoter P*petE* and the 5'UTR BCD2, harboring an insulated translation initiation feature[3]. Transcription terminates at the double terminator B0015 (rrnB T1/ T7Te). For improved

insulation of the P_{petE}:BCD2 expression module, B0015 and a *non-sense* 200-bp spacer[1] were ligated upstream of P_{petE}. (e) Sequence context of the P_{petE}:BCD2 expression module with feature annotation. (f) Sequences of features within the insulation and expression modules. KanR, kanamycin resistance cassette; SmR, streptomycin/spectinomycin resistance cassette; Rep, genes encoding replication proteins; *mobABC*: genes encoding mobilization proteins required for triparental mating; BB, biobrick; NB, neobrick; TSS, transcriptional start site. The figures were prepared with SnapGene Version 4.3.2 (https://www.snapgene.com).

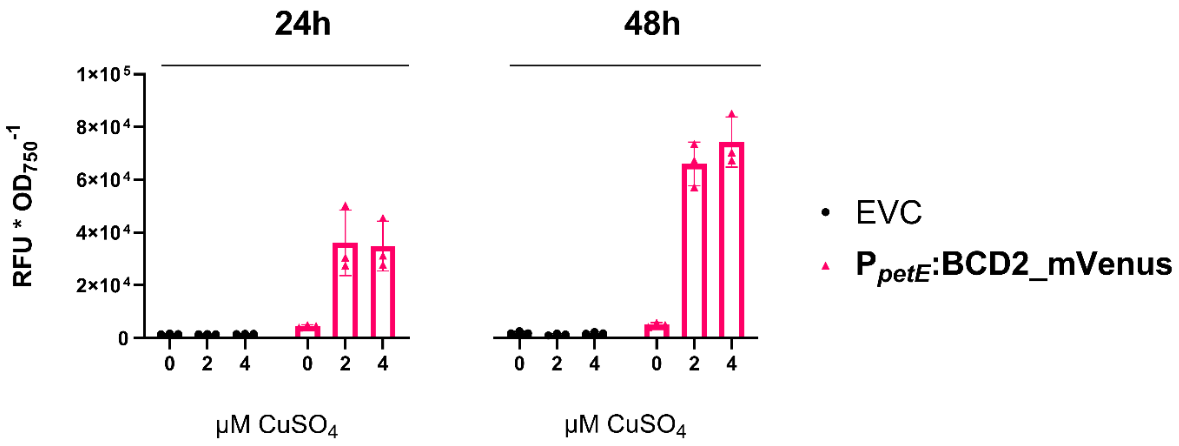

Figure S2: Quantitative assessment of expression modules P_{petE}:BCD2 upon copper treatment in *Synechocystis* 6803. The strain harboring pSHDY#_P_{petE}:BCD2:mVenus as well as an empty vector control strain (EVC) without fluorophor expression were pre-cultured in 24 well plates using each 600 µL BG11 (-Cu). At time point ”0h” cultures were diluted to OD ~0.1 and – each in triplicate - treated with 0, 2 and 4 µM $CuSO_4$, respectively. At time points 24h and 48h, each 200 µL were subjected to measurement of OD_{750} and mVenus fluorescence. At time point 24h the cultures were replenished with 200 µL of fresh BG11 including the corresponding $CuSO_4$ concentrations. Fluorescence data (RFU) were divided by the corresponding OD_{750} data [λ Ex./Em. 485/535nm * OD_{750}^{-1}] and are depicted as mean (bars) and single (dots) values with SD. Plasmid data are available as .dna files on Figshare: EVC (10.6084/m9.figshare.11473095), pSHDY#_P_{petE}:BCD2:mVenus (10.6084/m9.figshare.11473101). SD, standard deviation; OD, optical density; RFU, relative fluorescence units. The figure was prepared with Graphpad Prism 8.2.1. (https://www.graphpad.com/scientific-software/prism/).

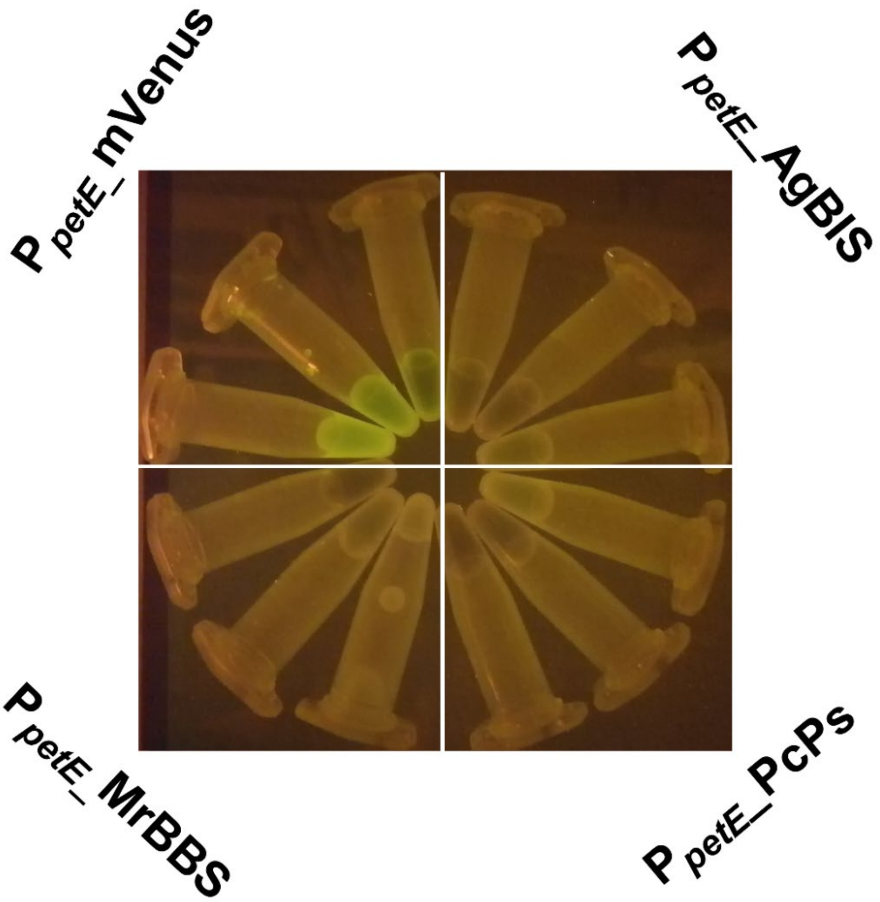

Figure S3: Fluorescence of *Synechocystis* strains at tp 192h of HDC. Aliquots of the cell suspensions were subjected to blue light (470 nm) illumination on a “Safeview-Mini2” transilluminator. The figures were processed using ImageJ software (https://imagej.nih.gov/ij/) and labelled with Graphpad Prism 8.2.1. (https://www.graphpad.com/scientific-software/prism/).

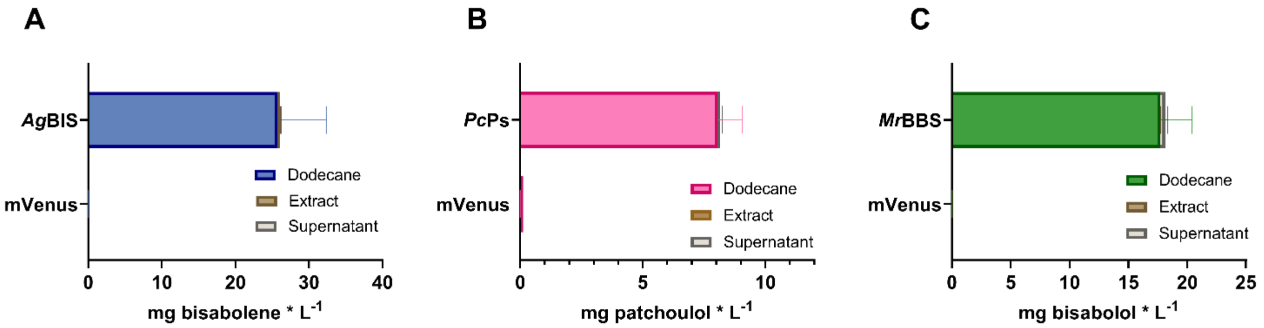

Figure S4: Volumetric partitioning of (a) bisabolene, (b) patchoulol and (c) bisabolol between dodecane, cellular extract and supernatant (growth medium) at tp 96h of HDC (run 2). All titer values refer to the volume of the growth medium (cf. Materials and Methods below). The figure was prepared with Graphpad Prism 8.2.1. (https://www.graphpad.com/scientific-software/prism/).

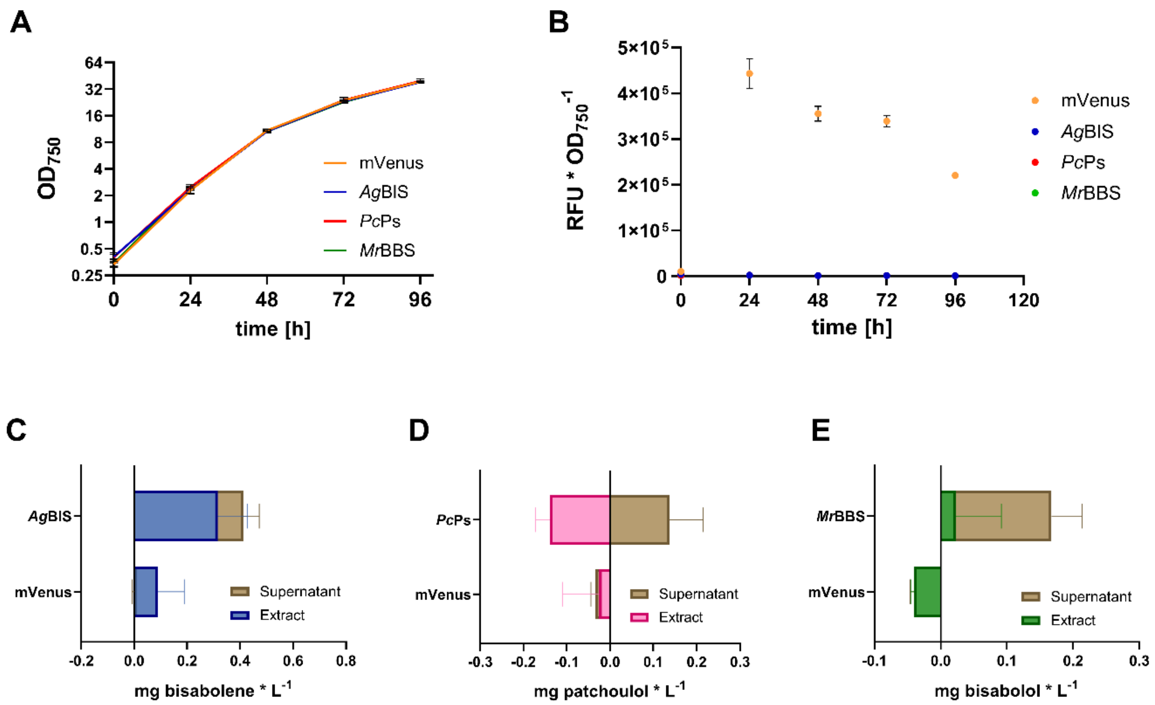

Figure S5: Sesquiterpenoid production and partitioning in HDC without dodecane overlay. Each 8 mL of culture shaken at 320 rpm (4 mm orbit) under constant multi-directional illumination with increasing light intensities. (a) Cell densities (OD_{750}) of strains P_{petE}_*Ag*BIS, P_{petE}_*Mr*BBS, P_{petE}_*Pc*Pc *and* P_{petE}_mVenus were recorded daily over a time period of 4 days. Induction with 4 µM CuSO4 was conducted at tp 0 and 48h. Data represent mean values from three independent cultures with SD. The y-axis scale is logarithmic (log2). (b) mVenus accumulation profile over time. Fluorescence data (RFU) were divided by the corresponding OD_{750} data [λ Ex./Em. 485/535nm * OD_{750}^{-1}] and are depicted as mean values with SD (cf. a). Volumetric partitioning of (C) bisabolene, (D) patchoulol and (E) bisabolol between cellular extract and supernatant (growth medium) at tp 96h of HDC. Apparent negative values of patchoulol (and bisabolol) titers result from slightly varying signal-to-noise ratios within the respective retention time frames. The figures were prepared with Graphpad Prism 8.2.1. (https://www.graphpad.com/scientific-software/prism/).

Material and Methods

Extraction and quantification of sesquiterpenoids from cell pellets and growth media

For initial fractionation of HDC cultures into cell pellets and media supernatant, each 4 mL of cell cultures from tp 96h of HDC were spun down for 10 min at 5000 g and 4 °C (Centrifuge 5804R, Rotor A-4-44, Eppendorf). The supernatants were transferred to fresh tubes and centrifuged again (10 min, 5000g, 4 °C). Each 3 mL of the supernatant were subjected to terpenoid extraction by adding 250 µL dodecane (incl. 2.5 µg * mL^{-1} BCP as IS and 0.05% butylated hydroxytoluene (BHT) as antioxidant); samples were vigorously vortexed for 15 sec and further agitated overnight in a horizontal position at ~90 rpm on an orbital shaker. The samples were centrifuged (10 min at 5000 g, RT) and each 200 µL of the dodecane overlay were subjected to GC-FID analysis (cf. main text).

Pellets were resuspended in fresh 1 mL thylakoid buffer (cf. main text). Equivalents of each 50 OD units were diluted in thylakoid buffer to a final volume of 400 µL. To correct for variations in the extraction efficiency, each 4 µL BCP (25 mg *mL^{-1}) were added as IS before extraction, which would correspond

to a final concentration of 2.5 µg * mL^{-1} BCP in the extraction solvent (dodecane), given that the efficiency equals 100%. A volume of ~200 µL 425-600 µm glass beads (acid-washed, 20-40 U.S. sieve, Sigma) was added and samples were homogenized using a Precellys24 tissue homogenizer (Bertin technologies) for 2 x 30 s at 5600 rpm with a 2 min interval. For extraction, each 400 µL dodecane (incl. 0.05% butylated hydroxytoluene (BHT) as antioxidant) were added; the samples were vigorously vortexed for 15 sec and agitated overnight in a horizontal position at ~90 rpm on an orbital shaker. The samples were centrifuged (5 min at 13000 g, RT) and each 200 µL of the dodecane overlay were subjected to GC-FID analysis (cf. main text).

Terpenoid yields were calculated based on GC-FID data as product titer in the culture [mg L^{-1}]. For peak area [µV·s] normalization individual values were divided by the corresponding IS peak area and multiplied with the mean IS value of all biological and ES samples.
Raw volumetric concentrations [µg * mL^{-1}] were calculated with the linear equation from ES calibration charts derived from the same GC cycle. For bisabolene, each value was further multiplied with factor 0.2752, to correct for impurities of the commercial standard.
The mean raw concentration of all blank samples from the same run were subtracted from each value. The total concentration in the supernatant extracts was extrapolated with the corresponding scale factor (e.g. (10 µg * mL^{-1} * 0.25/1000) = 0.00025 mg * 0.25 mL^{-1} extract). This value was divided by the actual culture volume and multiplied with factor 1000 to extrapolate the final product titer [mg * L^{-1}].
The total concentration in the cellular extracts was extrapolated with the corresponding scale factor (e.g. (10 µg * mL^{-1} * 0.4/1000) = 0.004 mg * 0.4 mL^{-1} extract). This value was multiplied with the the quotient of the individual dilution factor (i.e. normalization to OD 50 in 400 µL buffer) divided by the concentration factor 4 (pellet from 4 mL culture resuspended in 1 mL buffer), which was further multiplied with factor 10 (to convert µg * 400µL^{-1} extract to µg * 4 mL^{-1} culture Vol.).
Example:
(0.004 mg * 0.4 mL^{-1} extract) * individual dilution factor 1.25 = 0.05 mg * 0.4 mL^{-1} extract;
(0.05 mg * 0.4 mL^{-1} extract) / concentration factor 4 = 0.00125 mg * 0.4 mL^{-1} extract;
(0.00125 mg * 0.4 mL^{-1} extract) * factor 10 = 0.0125 mg * 4 mL^{-1} culture.
To extrapolate the final product titer [mg * L^{-1}], this value was eventually divided by 4 (mL culture sample volume), multiplied with factor 1000 (conversion of mg * mL^{-1} to mg * L^{-1}) and corrected for evaporation (multiplication with evaporation factor = actual Vol./ theoretical Vol.).
Example:
(0.0125 mg * 4 mL^{-1} culture)/ 4 = 0.003125 mg * mL^{-1} culture;
0.003125 mg * mL^{-1} extract * 1000 = 3.125 mg * L^{-1} culture;
3.125 mg * L^{-1} culture * (8 mL/ 6 mL) = 4.1666 mg * L^{-1} culture

1. Yeung, E. *et al.* Biophysical Constraints Arising from Compositional Context in Synthetic Gene Networks. *Cell Syst* **5**, 11-24 e12, doi:10.1016/j.cels.2017.06.001 (2017).
2. Mitschke, J. et al. An experimentally anchored map of transcriptional start sites in the model cyanobacterium *Synechocystis* sp. PCC 6803. *Proc Natl Acad Sci U S A* **108**, 2124-2129, doi:10.1073/pnas.1015154108 (2011).
3. Mutalik, V. K. *et al.* Precise and reliable gene expression via standard transcription and translation initiation elements. *Nat Methods* **10**, 354-360, doi:10.1038/nmeth.2404 (2013).
